# Lipoprotein metabolism in familial hypercholesterolemia

**DOI:** 10.1016/j.jlr.2021.100062

**Published:** 2021-03-03

**Authors:** Kévin Chemello, Javier García-Nafría, Antonio Gallo, Cesar Martín, Gilles Lambert, Dirk Blom

**Affiliations:** 1 Inserm UMR 1188 DéTROI, Université de La Réunion, Saint- Denis de La Réunion, France; 2 Institute for Biocomputation and Physics of complex systems (BIFI), University of Zaragoza, Zaragoza, Spain; 3 Laboratorio de Microscopías Avanzadas, University of Zaragoza, Zaragoza, Spain; 4 Cardiovascular Prevention Unit, Department of Endocrinology and Metabolism, Pitié-Salpêtrière University Hospital, Paris, France; 5 Laboratoire d'imagerie Biomédicale, INSERM 1146, CNRS 7371, Sorbonne University, Paris, France; 6 Instituto Biofisika (UPV/EHU, CSIC) and Departamento de Bioquímica, Universidad del País Vasco UPV/EHU, Bilbao, Spain; 7 Hatter Institute for Cardiovascular Research in Africa and Division of Lipidology, Department of Medicine, University of Cape Town, Cape Town, South Africa

**Keywords:** familial hypercholesterolemia, lipoproteins, LDL-C, CVDs, lipoprotein (a), ANGPTL3, angiopoietin-like 3, apo(a), apolipoprotein (a), apoA1, apolipoprotein A1, apoB, apolipoprotein B, apoB100, apolipoprotein B100, ARH, autosomal recessive hypercholesterolemia, ASCVD, atherosclerotic CVD, FCR, fractional catabolic rate, FDB, familial defective apolipoprotein B, FH, familial hypercholesterolemia, GOF, gain-of-function, HeFH, heterozygous familial hypercholesterolemia, HoFH, homozygous familial hypercholesterolemia, JELIS, Japan EPA Lipid Intervention Study, LDLR, LDL receptor, LDLRAP1, LDLR adapter protein 1, Lp(a), lipoprotein (a), PCSK9, proprotein convertase subtilisin kexin type 9, REDUCE-IT, Reduction of Cardiovascular Events with Icosapent Ethyl-Intervention Trial, TESLA, Trial Evaluating PCSK9 Antibody in Subjects With LDL Receptor Abnormalities, TG, triglyceride, TRL, TG-rich lipoprotein

## Abstract

Familial hypercholesterolemia (FH) is one of the most common genetic disorders in humans. It is an extremely atherogenic metabolic disorder characterized by lifelong elevations of circulating LDL-C levels often leading to premature cardiovascular events. In this review, we discuss the clinical phenotypes of heterozygous and homozygous FH, the genetic variants in four genes (*LDLR/APOB/PCSK9/LDLRAP1*) underpinning the FH phenotype as well as the most recent in vitro experimental approaches used to investigate molecular defects affecting the LDL receptor pathway. In addition, we review perturbations in the metabolism of lipoproteins other than LDL in FH, with a major focus on lipoprotein (a). Finally, we discuss the mode of action and efficacy of many of the currently approved hypocholesterolemic agents used to treat patients with FH, with a special emphasis on the treatment of phenotypically more severe forms of FH.

Familial hypercholesterolemia (FH) is an inherited metabolic disease associated with high levels of circulating LDL-C and premature CVD (1). Heterozygous familial hypercholesterolemia (HeFH) is a common genetic disorder resulting from an autosomal dominant or codominant inheritance pattern with an estimated prevalence of 1 in 250 subjects in most countries. However, the prevalence is much higher in regions or localized populations with founder effects (2, 3). Homozygous familial hypercholesterolemia (HoFH) is characterized by a much lower prevalence, around one case in 160,000–300,000 subjects (4). In rare instances, HoFH is transmitted as a recessive trait (5). Here, we focus on the clinical phenotypes of FH, the genetic variants at the origin of the phenotype as well as on the most recent experimental approaches used to investigate molecular defects affecting the LDL receptor (LDLR) pathway in FH. The perturbations of lipoprotein metabolism beyond LDL as well as the mode of action and efficacy of the currently approved hypocholesterolemic agents used to treat patients with FH are also reviewed.

## Clinical presentation and diagnosis

Untreated, FH frequently results in premature atherosclerotic CVD (ASCVD), the first ASCVD in HoFH often occurring in childhood or adolescence (6), whereas patients with HeFH usually experience their first ASCVD event in the third or fourth decade of life (7). Lifelong exposure to high LDL-C levels has been shown to be the main determinant of the increased risk of ASCVD in patients with FH (3, 4, 8, 9). The coronary territory is by far the most affected (10, 11, 12), but cerebrovascular and/or peripheral artery diseases are also seen in some patients with FH (13, 14).

A high untreated LDL-C is often the first clue alerting clinicians to a possible diagnosis of FH. After exclusion of secondary causes of hypercholesterolemia, many clinicians use scoring systems that incorporate both clinical and laboratory criteria to assist in the diagnosis of FH. The Simon-Broome criteria algorithm takes into account the proband total and LDL-C, the presence of tendon xanthomata, the presence of a genetic mutation, and a family history of ASCVD (7). The Dutch Lipid Clinics Network Score is probably the most well known of such scores. It considers the same criteria with a more refined classification for LDL-C level ranges as well as for family history of FH and/or premature ASCVD and genetic analyses (15). The *International Classification of Diseases, Tenth Revision* definition algorithm takes into account LDL-C levels and the eventual presence of a mutation in the index case patient and relatives. In a similar way, make early diagnosis to prevent early deaths criteria take into account the proband age, his and/or her LDL-C, and the closest parental degree of a confirmed affected relative (16).

The association of common cardiovascular risk factors, such as male sex, smoking, hypertension and diabetes, as well as low HDL-C in patients with FH increases exponentially the cardiovascular morbidity and mortality and accounts for about one in four-to-five ASCVD cases within this population (17, 18). Likewise, the presence of metabolic disorders such as insulin resistance in obesity and diabetes has shown to further increase the risk of ASCVD in FH. The prevalence of obesity has been reported at around 20% in several FH cohorts, whereas a more variable estimation of type 2 diabetes has been shown across the world, ranging from 1.75% to 25% (12, 19, 20, 21, 22). Patients with FH have been found in a number of studies to exhibit lower prevalence of diabetes compared with their unaffected siblings (23).

## Genetics and functional characterization of FH

**Fig. 1:**
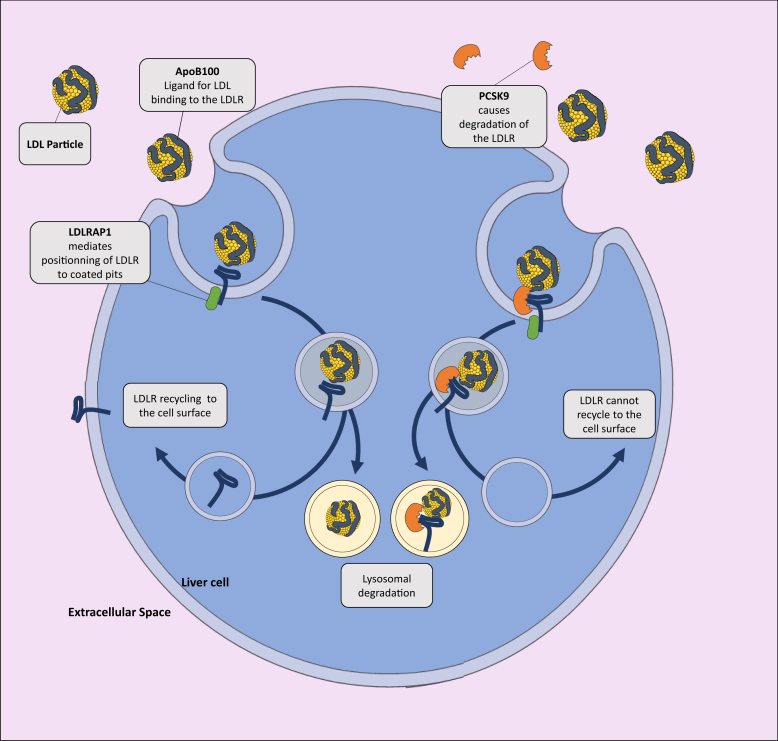
Schematic overview of the LDL receptor (LDLR) pathway. The extracellular domain of the LDLR binds to apolipoprotein B100 (apoB100) of an LDL particle. The intracellular domain of the receptor interacts with its adaptor protein [LDLR adapter protein 1 (LDLRAP1)], allowing endocytosis of LDL particle into clathrin-coated vesicles. The LDL-LDLR complex reaches the endosome where the acidic pH induces the dissociation of the complex. The receptor is recycled back to the cell surface, whereas the particle is degraded in the lysosomal compartment. When proprotein convertase subtilisin kexin type 9 (PCSK9) is bound to the receptor, the LDL-LDLR complex cannot dissociate, and the receptor ends up in the lysosome where it is degraded.

The genetic defects underlying FH reside either on the *LDLR*, *APOB*, *PCSK9*, or *LDLRAP1* genes and result in reduced clearance of plasma LDL by the LDLR pathway leading to lifelong elevations in circulating LDL-C levels (Fig. 1).

### The LDLR

**Fig. 2:**
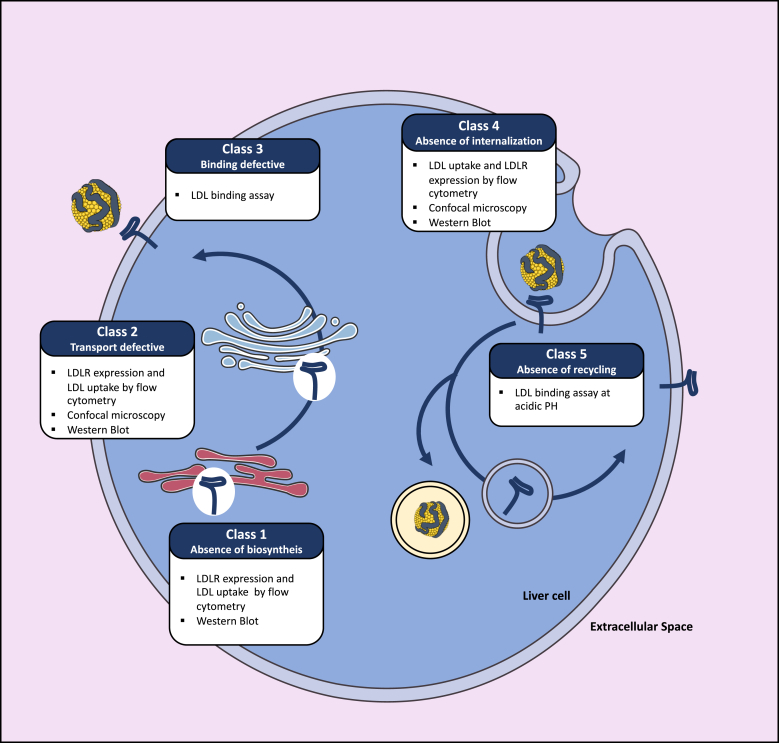
Assessment of LDL receptor (LDLR) class defects. The pathogenicity of LDLR variants results either from an absence of biosynthesis of the receptor (class 1), a transport defect of the receptor from the endoplasmic reticulum to the Golgi (class 2), poor binding of the receptor to LDL particles (class 3), an absence of receptor internalization (class 4), or failure to recycle back to the cell surface (class 5).

In approximately 90% of the cases, FH results from the presence of mutations in the *LDLR* gene itself (3, 24). More than 1,700 different *LDLR* mutations have been described (25, 26). LDLR mutations can either result in an absence of biosynthesis (class 1 defects), preclude the maturation/transportation of the receptor from the endoplasmic reticulum to the Golgi (class 2), reduce the affinity of the receptor for LDL particles (class 3), alter the internalization of the receptor/ligand complex (class 4), or prevent normal recycling of the LDLR back to the cell surface (class 5) (27) (Fig. 2).

The LDLR pathway was discovered in 1974 by Brown and Goldstein. They showed that the high affinity of ^125^iodine-radiolabeled LDL for human dermal fibroblast was absent when fibroblasts were obtained from patients with HoFH (1, 28). Since these pioneering studies, novel approaches have been successfully developed to determine LDLR activity and hence the pathogenicity of *LDLR* genetic variants ex vivo. For instance, lymphocytes isolated from patients carrying *LDLR* variants and subsequently immortalized have progressively replaced dermal fibroblasts when studying LDLR function (29, 30). Primary lymphocytes can alternatively be expanded in culture using either mitogens or CD3/CD28 dynabeads (31, 32). In addition, LDLR expression can be enhanced by serum deprivation or statin treatment (33, 34), thus facilitating the assessment of LDLR expression by Western blot, as well as the characterization of LDLR activity by flow cytometry using fluorescently labeled LDL (35). These novel approaches yield qualitatively and quantitatively similar results to those obtained in the past using dermal fibroblasts and radiolabeled LDL. Labeling of the LDLR with fluorescent antibodies and LDL particles with fluorescent dyes, respectively, allows the determination of LDLR expression levels at the cell surface and the assessment of LDL cellular binding at 4°C as well as cellular uptake at 7°C. Trypan blue is used in these experiments to quench the fluorescence of noninternalized LDL particles (35).

Nowadays, most LDLR functional studies can easily be carried out with the LDLR-deficient Chinese hamster ovary cell line ldlΔ7 transfected with plasmids allowing the expression of LDLR variants (35, 36). Cellular LDLR expression is assessed by Western blot, which allows the detection of the LDLR precursor (120 kDa) and of the mature receptor (160 kDa). LDLR cell surface expression and LDLR activity (i.e., LDL binding and uptake) are assessed by flow cytometry, as described above. In addition, confocal microscopy analyses permit the determination of most class-type defects of LDLR variants by assessing the colocalization of the receptor with endosomal, lysosomal, and/or endoplasmic reticulum-specific markers. A modified ELISA binding assay measuring the affinity of LDLR variants for LDL particles is however more suited to detect a class 3 defect (37). A class 5 defect can be determined by performing a similar binding assay at the acidic pH found in endosomes (38). These in vitro approaches are easy to set up as there is no need for clinical samples. They also allow accurate determination of the mechanisms underlying the pathogenicity of each LDLR variant, as they mimic HoFH conditions that can be masked in heterozygous states, in particular for mildly pathogenic LDLR variants.

### Apolipoprotein B100

**Fig. 3:**
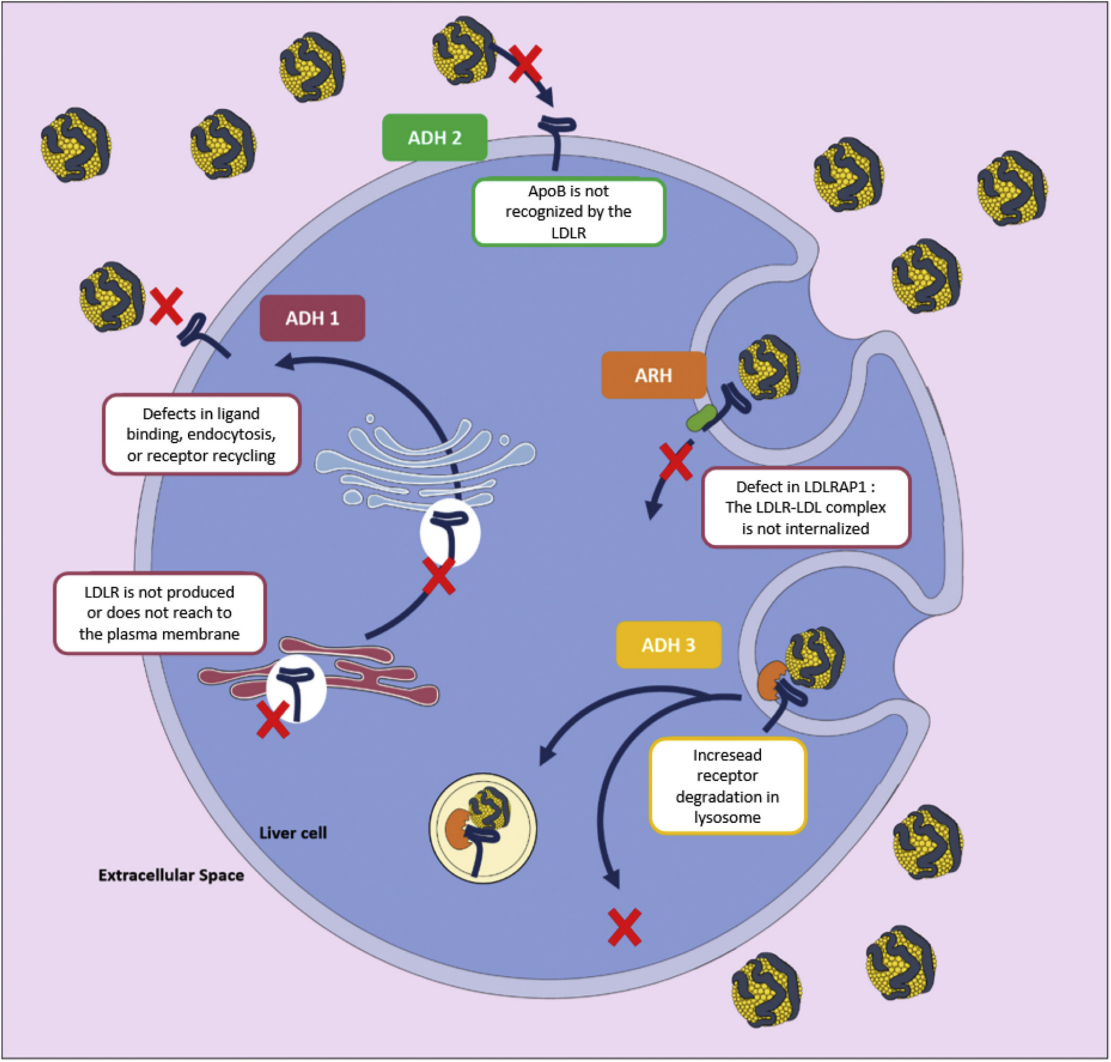
Molecular causes of familial hypercholesterolemia. Autosomal dominant hypercholesterolemia (ADH) is caused by mutations either on the *LDLR* (ADH1), *APOB* (ADH2), or *PCSK9* (ADH3) genes. Biallelic mutations on *LDLRAP1* promote autosomal recessive hypercholesterolemia (ARH).

In approximately 5% of the cases, FH results from the presence of mutations on apolipoprotein B100 (apoB100), the major protein component of LDL that serves as a ligand for the LDLR (39). This condition is also named familial defective apolipoprotein B (FDB) (Fig. 3). Only a handful of *APOB* mutations causing FH have been reported, and they are all located within (or in the vicinity of) the LDLR binding region of apoB100 (26).

Initially, LDL binding and LDL uptake studies using radiolabeled LDL isolated from patients with APOB genetic variants were used to assess their pathogenicity (39). Fluorescently labeled LDL isolated from patients is now used to perform these assays (40, 41), either in primary human lymphocytes or in cell lines expressing wild-type LDLR, as described above. The U937 cell line that derives from a histiocytic lymphoma has been extremely helpful in that respect. U937 cells lack 3-ketosteroid reductase, required for endogenous cholesterol synthesis, and therefore require extracellular cholesterol supply for proliferation (42). The proliferation rate of these cells in the presence of LDL carrying wild-type or FDB variants is a measure of the ability of the LDL to bind to the LDLR (38, 40, 41). A modified ELISA binding assay using recombinant LDLR for capture of wild-type or FDB LDL can also be used (43).

### Proprotein convertase subtilisin kexin type 9

In approximately 1% of cases, FH results from the presence of “gain-of-function” (GOF) mutations on proprotein convertase subtilisin kexin type 9 (PCSK9) (44). PCSK9 is a protein secreted by the liver. At the cell surface, PCSK9 binds to the first epidermal growth factor-like repeat homology domain of the LDLR. After endocytosis, the affinity between the LDLR and PCSK9 is much higher (as a result of the acidic pH conditions of endosomes), and the interaction between PCSK9 and LDLR locks the receptor in an extended or “open” conformation (45) (Fig. 1). The failure of the receptor to adopt a “closed” conformation in the endosome precludes normal recycling to the plasma membrane and targets the LDLR for lysosomal degradation (Fig. 3). PCSK9 has also been shown to promote LDLR decay via an intracellular route. Similar to *LDLR* gene defects, *PCSK9* GOF mutations lower the abundance of the LDLR at the cell surface in many different ways (46, 47). For instance, genetic variants on PCSK9 can display either higher transcriptional activity, or resistance to cleavage by furin, or increased affinity for the LDLR.

The methodologies to characterize PCSK9 GOF variants are quite heterogeneous and rely on different approaches, such as immunocytochemistry, flow cytometry, and biochemistry techniques (48, 49, 50, 51). As for patients with FDB, fibroblasts or lymphocytes from PCSK9 mutation carriers should be evaluated to ascertain that their LDLR is expressed normally and fully functional to rule out any potentially undetected LDLR defect (40, 51). Then, the synthesis, secretion, and LDLR inhibitory effects of PCSK9 variants can be comparatively assessed by transfecting cell lines that do not endogenously express PCSK9, such as human embryonic kidney 293 cells, with wild-type or PCSK9 variant expression vectors. Intracellular and secreted PCSK9 levels are determined by Western blot to assess the ratio of nonprocessed/processed PCSK9 in cell extracts, the levels of secretion of PCSK9 in the culture medium, and the ability of furin to mediate the cleavage of secreted PCSK9, comparatively for wild-type and PCSK9 variants. LDLR cell surface expression and fluorescently labeled LDL uptake are determined by flow cytometry as above.

Given that PCSK9 acts primarily as a secreted protein, the extracellular activity of wild-type and PCSK9 variants can be comparatively assessed by adding the recombinant PCSK9 proteins in the culture medium of hepatoma cell lines (e.g., HepG2) prior to measurement of cell surface LDLR expression and fluorescent LDL cellular uptake by flow cytometry (47, 50, 51, 52). Another valuable parameter that provides important information about PCSK9 variants is to measure their affinity for the LDLR at the cell surface and in endosomes. This can be evaluated by solid-phase immunoassay at pH 7.4 and 5.2, respectively, using the recombinant LDLR ectodomain to capture PCSK9 (47, 51). Given that some PCSK9 variants have been shown to inhibit LDLR prior to secretion, this intracellular activity can be assessed in human embryonic kidney 293 cells coexpressing the LDLR ectodomain and either wild-type or PCSK9 variants. The amount of LDLR ectodomain secreted in the culture medium can be assessed by Western blot and recapitulates the ability of PCSK9 variants to impact the translocation of the LDLR from intracellular compartments to the cell surface (47, 52).

### The LDLR adaptor protein 1

The HoFH phenotype can also be caused by variants in the LDLR adapter protein 1 (*LDLRAP1*) gene, but this particular condition is an autosomal recessive disorder known as autosomal recessive hypercholesterolemia (ARH). Heterozygous carriers of *LDLRAP1* mutations present with normal circulating lipoprotein levels (53). LDLRAP1 bridges the intracellular domain of the LDLR with clathrin, an essential protein involved in the formation of endocytic vesicles and hence LDL cellular uptake (Figs. 1 and 3).

Ex vivo investigation of ARH is not as straightforward as that of autosomal dominant hypercholesterolemia. For instance, LDLRAP1 functionality cannot be assessed in patient's dermal fibroblasts, as the adaptor protein is not required for LDLR endocytosis into clathrin-coated pits in this particular cell type (54, 55, 56). In contrast, LDLRAP1 is essential for LDLR internalization into human lymphocytes and hepatocytes. The cellular assessment of LDLRAP1 variants can therefore be performed in ARH lymphocytes, where cell surface LDLR levels are always much higher than in control lymphocytes, whereas fluorescent LDL uptake is significantly reduced (57).

### An extreme phenotypic variability

The clinical phenotype of patients with FH may vary considerably. The extent of LDL-C elevation is the most important determinant of phenotypic severity. LDL-C elevation is not only related to a gene-dosage effect (the presence of two mutations instead of one is associated with a more severe phenotype) but also depends on the functional impact of mutations. For instance, *LDLR* mutations are usually described as “null” (<2% of normal LDLR activity) or “defective” (between 2% and 25% of normal activity) (4). Null mutations correlate with the more severe forms of HeFH. The following sequence of genotypes is associated with the most severe to mildest phenotypes: homozygosis for LDLR null mutations; compound heterozygosis for LDLR null and LDLR-defective mutations; homozygosis for LDLR-defective mutations or LDLRAP1; homozygosis for defective APOB or PCSK9 GOF mutations; and heterozygosis for LDLR null mutations. The FH phenotype is also modulated by other genetic and environmental factors, and patients with identical mutations also show marked phenotypic variability. The mildest forms of HoFH often overlap with more severe forms of HeFH; some HeFH in turn may overlap with more severe forms of polygenic FH.

## Lipoprotein metabolism in FH beyond LDL

Although impaired LDLR function, and thus decreased clearance of LDL from the circulation, is the hallmark of FH, decreased LDLR function does not entirely explain the dyslipidemia seen in FH. The mutational diversity in these four genes variably modulates the LDLR pathway and thereby determines the heterogeneity of LDL-C levels found in FH. Although the LDLR is expressed in many cell types, the liver is by far the primary site of LDL cellular uptake, which is evidenced by the report of an accidental transmission of a severe FH phenotype to a previously normolipemic liver transplant recipient (58). Compared with LDL, the circulating levels of other lipoproteins are not or mildly affected in FH, with some exceptions in particular when a metabolic syndrome is present. However, the levels of lipoprotein (a) [Lp(a)] appear to be increased in patients with FH compared with the general population.

### Lipoprotein (a)

Lp(a) is an atherogenic lipoprotein consisting of an apolipoprotein (a) [apo(a)] protein covalently tethered to the apoB100 of an LDL particle. Apo(a) is encoded by the *LPA* gene and presents a highly repetitive structure, the kringle IV2 domain present in 1 to more than 40 copies per allele. The size of apo(a) explains up to 70% of Lp(a) variance in humans: the number of KIV2 domains on apo(a) is inversely proportional to Lp(a) plasma levels. The initial studies that have investigated Lp(a) in FH have not yielded conclusive results, given the wide variation of Lp(a) resulting from the size polymorphism of apo(a) (59). However, the assessment of FH and non-FH siblings with apo(a) isoforms identical by descent has clearly demonstrated that Lp(a) is approximately twice higher in patients with FH than in their nonaffected family members (59). FH homozygotes with two nonfunctional LDLR alleles also display 2-fold higher Lp(a) levels than their heterozygote relatives (60). Likewise, patients with FDB have higher Lp(a) than non-FDB family members (61), and PCSK9 GOF mutation carriers also similarly display higher Lp(a) than non-FH controls (62). Although these combined results appear to advocate for a direct role of the LDLR in mediating Lp(a) plasma clearance, no such conclusion was drawn from these studies by their authors.

For instance, in vitro, the binding and cellular uptake of Lp(a) is reduced in primary HoFH dermal fibroblasts totally lacking the LDLR in some studies but not in others (63, 64). We recently reported that the cellular uptake of Lp(a) was similar in primary lymphocytes isolated from patients with HoFH and healthy donors (65). In vivo, the pharmacological modulation of the LDLR using PCSK9 inhibitors did significantly affect neither the fractional catabolic rate (FCR) of Lp(a) in nonhuman primates (66) nor the hepatic capture of fluorescently labeled Lp(a) in liver humanized mice (65). In humans, the FCR of Lp(a) was not statistically different between control individuals and HeFH or HoFH patients separately, but compared with non-FH controls, the FCR of Lp(a) was significantly reduced when combining all patients with FH (60, 67). In patients, enhancing LDLR function using PCSK9 inhibitors in monotherapy nonsignificantly increased the FCR of Lp(a) in one study (68) but reduced it in another study (69). Importantly, unlike PCSK9 inhibitors, statins that also increase the abundance of the LDLR significantly raise Lp(a) in humans (70).

**Fig. 4:**
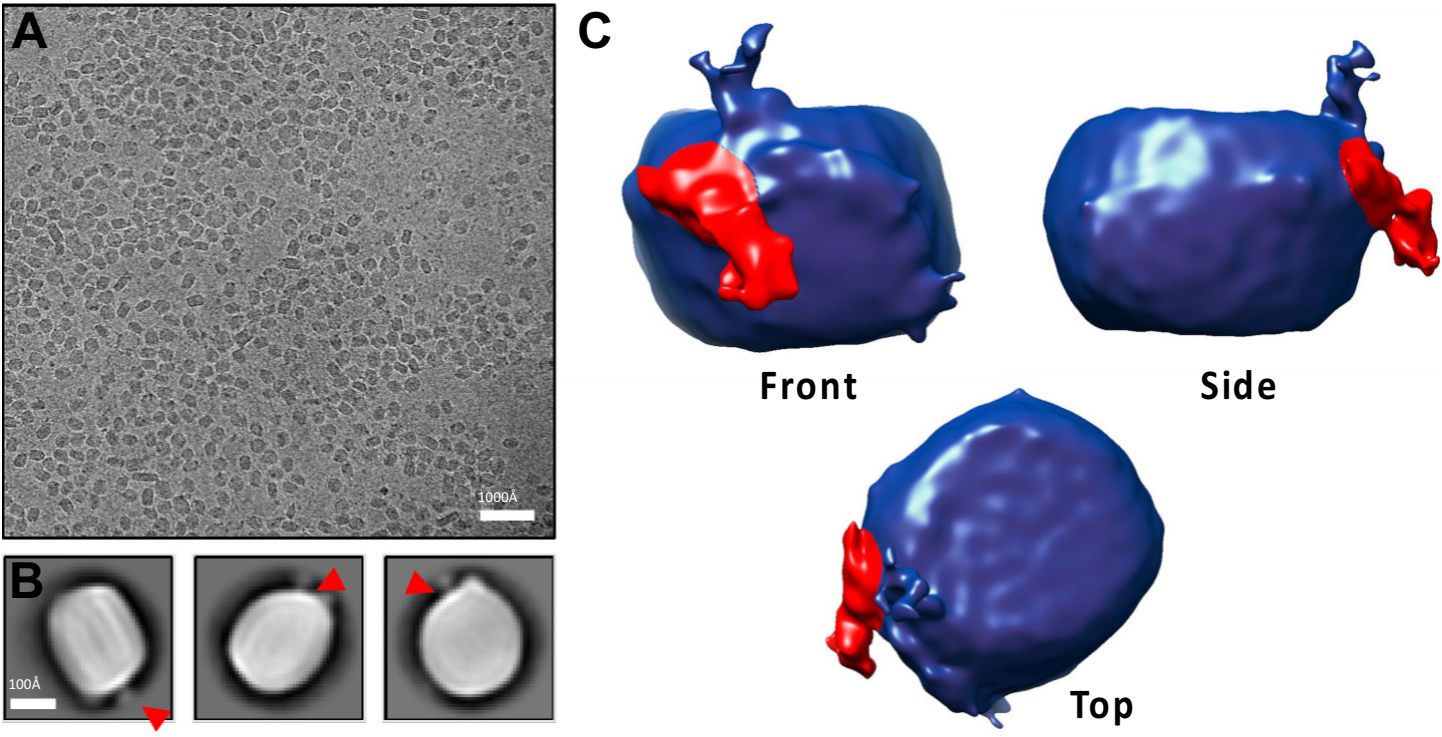
3D reconstruction of lipoprotein (a) [Lp(a)] particles by cryogenic electron microscopy. Lp(a) particles (0.8 mg/ml) were vitrified using glow discharged Cu grids 1.2/1.3, and an FEI Vitrobot IV and 900 micrographs were collected (1.65 Å/pixel, total dose 50e^−^/A^2^). Images were motion and contrast transfer function corrected following particle picking, 2D averaging, ab initio 3D reconstruction, 3D classification, and refinement. Raw micrographs showed characteristic LDL-like particles (panel A), and 2D averages displayed weak density protrusions from the surface of those particles corresponding to their apo(a) moieties (panel B, arrowheads). A 3D reconstruction (with 42,247 particles at a 15 Å resolution) showed additional cryogenic electron microscopy density (in red) corresponding to the insertion point of apo(a) (panel C).

Apo(a) isoforms have been reported to vary from 300 to 800 kDa in size (71), and recombinant apo(a) (containing 17 kringle IV domains) has been shown to extend up to 800 Å into solution. Hence, to gain insights into the determinants of Lp(a) clearance, Lp(a) has been subjected to diverse structural studies over the years, however, without reaching a consensus. Atomic force microscopy suggested that apo(a) is anchored to the LDL by the N and C terminus. Small-angle X-ray scattering suggested that apo(a) locates to the surface and wraps around the LDL particle (72), and studies using electron microscopy concluded that the bulk of apo(a) extends away from the LDL surface (73). To visualize Lp(a) particles in a near-native environment and at higher resolution, we recently purified LDL and Lp(a) particles and subjected them to cryogenic electron microscopy. The 2D images of these lipoproteins were averaged (Fig. 4A) and reconstructed to generate a tridimensional model (74). Unlike LDL, Lp(a) displays a weak density protrusion from the surface corresponding to the apo(a) moiety (Fig. 4B). The 3D model shows additional cryogenic electron microscopy density on Lp(a) particles corresponding to the insertion point of apo(a) (Fig. 4C). This feature was absent from human LDL (75). Apo(a) seems to adopt a disordered conformation, but it clamps to apoB100 in the vicinity of its LDLR binding site (75), which may cause steric hindrance preventing proper Lp(a) uptake by the LDLR.

The fact that Lp(a) is higher in patients with FH has recently been challenged by two independent studies. In 46,200 individuals from the Copenhagen General Population Study in whom Lp(a) was measured, mean Lp(a) concentrations were 23 mg/dl in individuals unlikely to have FH, 32 mg/dl in subjects with possible FH, and 35 mg/dl in those with probable or definite FH, based on the Dutch Lipid Clinics Network diagnostic criteria (76). However, after adjusting LDL-C levels for Lp(a) cholesterol to more accurately assess the FH status, those values were similar at 24, 22, and 21 mg/dl, respectively. Similar observations were made when using the make early diagnosis to prevent early deaths or Simon-Broome FH diagnostic criteria (77) as well as in the British Columbia FH cohort using the Dutch Lipid Clinics Network criteria (78), indicating that a substantial proportion of patients clinically diagnosed with FH are in fact hyperlipoprotein(a)emic and not genuine FH.

In the British Columbia FH cohort, Lp(a) was found higher than in the general reference population, but there was no difference in Lp(a) plasma levels between carriers of *LDLR* or *APOB* pathogenic variants compared with noncarriers (78). The authors rather found that elevated Lp(a) levels in FH were linked to a 2-fold higher prevalence of a specific single nucleotide polymorphism (rs10455872) on the *LPA* gene associated with an average 64 mg/dl increase in circulating Lp(a) levels (79) in that cohort compared with reference populations, suggesting that there may be an ascertainment bias in the association between FH and elevated Lp(a) (78). They further investigated this hypothesis using whole exome sequencing by identifying 221 “true” FH patients (i.e., with pathogenic mutations on the *LDLR, APOB*, or *PCSK9* genes) out of 37,486 individuals in the UK Biobank, without prior knowledge of their clinical history. As anticipated, these 221 individuals had significantly higher LDL-C and apoB100 plasma levels than the 37,265 non-FH individuals, but both groups displayed similar circulating Lp(a) concentrations (78). It therefore appears that the phenotypic determination of FH based on scores without genotyping for a pathogenic allele on *LDLR*, *APOB*, or *PCSK9* or without adjusting LDL-C for Lp(a) logically enriches the FH population with patients with hyperlipoprotein(a)emia.

These novel insights therefore cast a doubt on the consensus that Lp(a) is elevated in FH. But given that elevated Lp(a) can only accelerate the occurrence and aggravate the magnitude of cardiovascular events in patients already at very high risk, it cannot be emphasized enough that Lp(a) concentrations should be systematically measured in FH not only for diagnostic accuracy but also to better manage an apparent resistance to statins in these patients.

### High density lipoproteins

HDL-C has been widely explored in FH. Equivocal results have been found regarding HDL-C levels in FH, some studies finding no difference with non-FH populations, others finding lower levels (80, 81, 82). Some studies have focused on HDL particle size and showed that HDL particles are smaller in FH and thus more atherogenic (80). For instance, impaired reverse cholesterol transport has been shown to further increase cardiovascular risk (83) in both HeFH and HoFH, further underlining that HDL functionality, rather than mere cholesterol content of HDL, better reflects the atheroprotective functions of HDL particles (84). Thus, the centripetal transport of cholesterol from peripheral cells to feces appears altered in FH. The efflux of cellular cholesterol to HDL is apparently lower when HDL are isolated from patients with FH, leading to decreased esterification by LCAT, which is associated with an increased risk of ASCVD in a study conducted in 71 patients with HeFH and 66 normolipidemic individuals (85). The established antioxidant and anti-inflammatory properties of HDL also appear to be impaired in FH (86). Likewise, kinetics studies using stable isotope have shown a reduced turnover for apolipoprotein A1 (apoA1; both production and catabolism) in patients with FH (87). In HoFH, the impact of LDLR expression on HDL function has not been clearly established. ApoA1 FCR is reduced in HoFH suggesting an impaired transfer of cholesteryl esters to LDL. In addition, HDL size and composition appear impaired in subjects with FH and parallel an increased transfer of cholesteryl ester to LDL.

### Triglycerides

The presence of low HDL concomitant with high triglyceride (TG) levels usually mirrors an impaired metabolic status (e.g., insulin resistance, central obesity, diabetes). Thus, markers of insulin resistance such as low adiponectin levels, further increase the cardiovascular risk associated with FH (88). In this case, postprandial dyslipoproteinemia characterized by a hepatic overproduction of TGs as well as an impairment in the catabolism of TG-rich lipoproteins (TRLs) may be observed. Apart from the additive effect of the concomitancy of cardiometabolic risk factors in FH, the genetic defect underlying this condition may by itself impact on the metabolism of TRLs. Interactions between TRLs and the LDLR have been shown in animal models. Defects in LDLR function appears to also alter the interaction between apolipoprotein B (apoB) and apolipoprotein E, present on TRLs, and the LDLR thus determining a predisposition to postprandial hyperlipidemia in FH as well (89, 90).

In addition, the impairment of LDLR expression appears to influence the hepatic secretion of apoB-containing lipoproteins. Thus, patients with HoFH and animal models totally lacking LDLR activity display higher VLDL, intermediate density lipoprotein, and apoB production rates than non-FH individuals (reviewed in detail elsewhere (91, 92)). Similarly, PCSK9 GOF mutation carriers exhibit higher production rates of apoB-containing lipoproteins (93). For this reason, PCSK9 was also hypothesized to induce changes in ApoB48 metabolism in subjects with FH, but a study conducted in patients with 118 HeFH and 6 HoFH failed to establish a relationship between ApoB48 circulating levels and PCSK9 (94).

Given the aforementioned important aspects of impaired lipoprotein metabolism beyond LDL, patients with FH should clearly be monitored for HDL-C, TGs, apoA1, and apoB100 plasma levels more frequently.

## Lipid-lowering treatments

In FH, elevated levels of LDL-C are the main driver of atherosclerosis, and lowering LDL-C is the primary focus of pharmacological therapy. The cholesterol-year score integrates the exposure of the vasculature to cholesterol over time. The more severe the baseline LDL-C elevation is, the earlier and more intensive therapy is required (95). Generally, treatment should be commenced at the age of 8–10 years and immediately following diagnosis in children with HeFH and HoFH, respectively (3, 4, 96). The ultimate goal of treatment is to prevent clinical manifestations of ASCVD in patients with FH, rather than only delaying the first cardiovascular event.

There are no double-blind placebo-controlled cardiovascular outcome trials specifically targeting patients with FH, and it is unlikely that there ever will be. However, multiple observational studies support the benefit of treating patients with FH. In a retrospective study of a large cohort of Dutch patients with FH seen either before or after the availability of statin-based lipid-lowering therapy (using January 1990 as the cut-off date), the risk of a first cardiovascular event was 76% lower in patients with FH treated with a statin (97). In another retrospective review of patients with HeFH, moderate- to high-intensity statin therapy lowered the risk for CAD and mortality by 44% (98). Similarly, treatment initiation in children with HeFH slowed the rate of progression in carotid intima media thickness and was associated with a marked improvement in cardiovascular event free survival compared with their affected parent (99). In patients with HoFH, access to statin therapy was also associated with improved survival, with on-treatment LDL-C being the major determinant of outcome (9, 100).

Conceptually, there are three main mechanisms by which circulating LDL-C levels can be reduced. LDL clearance can be increased either by upregulating the number of LDLRs on the cell surface or by mechanically removing circulating LDL through lipid apheresis or plasma exchange. Lipid apheresis will not be discussed further, as it is likely that the introduction of novel therapies will continue to diminish the role of and requirement for such procedures. Decreasing the hepatic production and secretion of apoB-containing lipoproteins (primarily VLDL) also ultimately lowers circulating LDL. Most lipid-lowering therapies routinely used in the management of FH act predominantly by upregulating LDLR expression. These therapies work well in patients with HeFH who have one wild-type *LDLR* allele. In patients with HoFH, the efficacy of treatments that act by upregulating LDLR is determined in part by the residual LDLR function, and most patients with HoFH are not as responsive to such therapies as patients with other forms of hypercholesterolemia.

### Statins

Statins are the backbone of lipid-lowering therapy in patients with FH. Given their high baseline LDL-C, most adult patients require high doses of high-intensity statins (atorvastatin 40–80 mg/d or rosuvastatin 20–40 mg/d). Atorvastatin (80 mg/d) reduced LDL-C levels by 46–57% in patients with HeFH (101, 102). When rosuvastatin (40 mg/d) and atorvastatin (80 mg/d) were compared directly in a blinded and randomized forced titration study in patients with HeFH, they lowered LDL-C by 53.9% and 50.4%, respectively (103). Statin dosing should be individualized taking into account age, cardiovascular status, and LDL-C goal, as well as concomitant medication and tolerability. Statins are generally less effective in patients with HoFH, but the responses are highly variable. Patients with HoFH with biallelic *LDLR* null mutations often, but not always, fail to respond to such treatments (104, 105).

### Ezetimibe

Ezetimibe inhibits the absorption of cholesterol and phytosterols by enterocytes in the jejunal brush border by blocking the action of Niemann-Pick C1-Like 1 protein (106). Ezetimibe may also reduce biliary cholesterol reabsorption by hepatocytes through its interaction with Niemann-Pick C1-Like 1 protein in biliary canaliculi. Ultimately, ezetimibe depletes the hepatic steroid pool resulting in the upregulation of LDLR expression. In the Effect of Ezetimibe Plus Simvastatin Versus Simvastatin Alone on Atherosclerosis in the Carotid Artery trial, 720 patients with HeFH were randomized either to simvastatin (80 mg daily) with ezetimibe (10 mg daily) or simvastatin (80 mg daily) only following a single-blind 6-week placebo run-in period. The observed LDL-C reductions were −55.6% and −39.1%, respectively (107). In a study of patients with HoFH taking atorvastatin or simvastatin (40 mg) at baseline, randomization was either to uptitration of statin to 80 mg daily, addition of ezetimibe (10 mg daily) to an unchanged statin dose, or addition of ezetimibe (10 mg daily) and uptitration of the statin to 80 mg daily. LDL-C decreased by 6.7% with statin uptitration only, while addition of ezetimibe to any statin dose decreased LDL-C by 20.7%. Unfortunately, the study was not able to explore ezetimibe response as a function of residual LDLR function (108).

### PCSK9 inhibitors

Given its established role as a major inhibitor of the LDLR, PCSK9 has become a prime therapeutic target to lower LDL-C (45, 109). Currently, the two main approaches to decreasing the concentration of PCSK9 in the circulation are binding PCSK9 with fully human monoclonal antibodies (alirocumab or evolocumab) or inhibiting the hepatic synthesis of PCSK9 with a small interfering RNA such as inclisiran.

Alirocumab and evolocumab when added to preexisting lipid-lowering therapy in patients with HeFH lower LDL-C by an additional 50–60% (110, 111). With evolocumab, 63% (420 mg once a month) and 68% (evolocumab 140 mg once every 2 weeks) of patients were able to achieve LDL-C values below 1.8 mmol/l (111). The corresponding figures for alirocumab are 59.8% (Odyssey FH I) and 68.2% (Odyssey FH II) (110). In the Odyssey High FH study, which only enrolled patients with a baseline LDL-C above 4.1 mmol/l, 32.4% of patients lowered their LDL-C to 1.8 mmol/l or below (112). PCSK9 inhibition with monoclonal antibodies thus allows the majority of patients with HeFH to reach the <1.8 mmol/l LDL-C target for secondary prevention, although only a minority are able to reach the current target of <1.4 mmol/l for secondary prevention patients at very high risk of recurrent events. Monoclonal antibodies directed against PCSK9 are nowadays considered standard of care for patients with HeFH unable to reach target with statins ± ezetimibe, although funding difficulties still limit access in many parts of the world. In HeFH, the response to PCSK9 monoclonal antibodies is not influenced by the impact of the underlying *LDLR* mutation on LDLR function (residual or no residual activity) (111). This is because in patients with HeFH, upregulation of the wild-type *LDLR* allele likely accounts for most LDL lowering with a much smaller contribution from the mutated LDLR. However, individual responses to therapy may still differ markedly, even in individuals with identical mutations. The effect of PCSK9 inhibition with monoclonal antibodies on LDL-C in HoFH is even more variable. In the Rutherford 2 study, which enrolled patients with a clinical diagnosis of HeFH, 7 of the 331 participants were unexpectedly found to be genetic homozygotes or compound heterozygotes. Although the mean baseline LDL-C in these patients [5.3 mmol/l (SD 2.8)] was moderately higher than that of patients with HeFH with receptor-negative [4.4 mmol/l (1.3)] or receptor-defective mutations [3.9 mmol/l (1.0)], the LDL-C reductions at week 12 ranged from 48% (range, 38–64%) for evolocumab 420 mg once a month to 68% (range, 40–82%) for evolocumab 140 mg every 2 weeks. These responses are very comparable to those seen in HeFH receptor-negative patients; 61% reduction with evolocumab 140 mg every 2 weeks and 55% with evolocumab 420 mg administered monthly (111). Contrasting with this, the two receptor-negative patients enrolled in the Trial Evaluating PCSK9 Antibody in Subjects With LDL Receptor Abnormalities (TESLA) part A proof-of-concept study failed to respond to evolocumab despite plasma PCSK9 being lowered by more than 90% (113). Subsequently, the TESLA part B study confirmed the importance of residual LDLR function; patients with defective/defective mutation status had an overall better response (−31.8%; 95% confidence interval, −44.9 to −18.8) than patients with a defective/negative status (–21.0%; 95% confidence interval, –30.7 to –11.2) (114). The negative/negative patient in this study also showed no response to evolocumab. Once again, large variations in individual responses were seen, even in patients with two identical mutations. The TESLA part B study included eight patients who were genetic homozygotes for the c.681C > G *LDLR* mutation. The range of LDL-C reduction with evolocumab was 7.1–56.0% and correlated negatively with the number of LDLR expressed on their lymphocytes (115). Overall, monoclonal antibodies to PCSK9 reduce LDL-C by about half as much in homozygous compared with heterozygous patients. Residual LDLR functionality (determined by the type of mutation) and LDLR expression at the cell surface (determined by multiple factors) are important predictors of response.

Inclisiran lowered LDL-C by 39.7% (−47.9% difference from placebo) in 482 patients with a clinical diagnosis of HeFH. Next-generation sequencing of the four genes linked to FH identified 32 patients with either double HeFH, compound HeFH, or true HoFH. Baseline LDL-C in these patients was 3.9 mmol/l, somewhat lower than the baseline LDL-C in patients with definite pathogenic *LDLR* variants. The mean placebo-corrected LDL-C reduction in patients with two variants was 41.2%. LDL-C reductions achieved in patients with pathogenic, probably pathogenic, or variants of unknown significance were −46.0%, −48.3%, and −42.3%, respectively. Among patients with no identified causative mutation, the mean LDL-C reduction was 59.2% (116). Although *LDLR* mutations were classified somewhat differently in this study than in the Rutherford 2 study, which assessed functional impact (defective/negative) rather than the certainty of pathogenicity (pathogenic/probably pathogenic), both trials support the notion that in HeFH the type of mutation is of secondary importance in determining treatment response. The efficacy of inclisiran in patients with clinically diagnosed HoFH is likely to be similar to that of the monoclonal antibodies directed against PCSK9. Thus far, only the results of a small proof-of-concept study are available. In this study, inclisiran lowered LDL-C by 17.5–37.0% in three of four homozygous patients at day 180. The fourth patient not only failed to respond to inclisiran but also had a history of minimal responses to alirocumab and evolocumab.

### Mipomersen and lomitapide

Both mipomersen and lomitapide inhibit the production of apoB-containing lipoproteins and are therefore effective even in patients with no residual LDLR function. Mipomersen is an antisense oligonucleotide that binds the mRNA for apoB100, leading to its degradation and decreased hepatic synthesis of VLDL. Lomitapide inhibits microsomal TG transfer protein in the endoplasmic reticulum of enterocytes and hepatocytes reducing both chylomicron and VLDL production. Although both drugs are effective in patients with HeFH, their use is restricted to patients with HoFH because of their potential for significant hepatic toxicity. Mipomersen and lomitapide reduced LDL-C by a mean of 24.7 and 40% (intention-to-treat analysis) in their pivotal HoFH trials, respectively (117, 118). Increases in hepatic fat, with the development of hepatic steatosis in some patients are intrinsic to the mechanism of action of both drugs. Further research into the risk of hepatic steatosis progressing to steatohepatitis, fibrosis, and ultimately cirrhosis is ongoing. Adverse effects specific to each drug include injection site reactions and flu-like symptoms for mipomersen. Discontinuation rates were also high in the long-term extension studies of mipomersen. Lomitapide commonly causes gastrointestinal side effects, such as nausea, flatulence, and diarrhea. These adverse effects are most commonly seen early in treatment and can be mitigated by slow-dose titration and institution of a low-fat diet. Lomitapide remains a useful additional therapeutic option in homozygous patients who fail to respond adequately to statins, ezetimibe, and PCSK9 inhibition but should only be prescribed by physicians experienced in its use and willing to perform the mandated regular monitoring of hepatic function.

### Evinacumab

Evinacumab is a monoclonal antibody directed against angiopoietin-like 3 (ANGPTL3). ANGPTL3 is an inhibitor of endothelial and lipoprotein lipases. Loss-of-function mutations in ANGPTL3 cause familial combined hypolipidemia, a condition first identified in the small Italian town of Campodimele and characterized by low levels of both apoB- and apoA1-containing lipoproteins (119). In a trial of patients with refractory hypercholesterolemia, defined as an LDL-C of greater than 1.8 mmol/l or greater than 2.6 mmol/l for those with or without clinical atherosclerotic disease despite treatment with maximally tolerated statin (with or without ezetimibe) and a PCSK9 monoclonal antibody, evinacumab given in varying doses either subcutaneously or intravenously reduced LDL-C in a dose-dependent manner by 24.2–56.0% compared with placebo. In this trial, 72% of patients had FH (120).

In patients with HoFH, evinacumab reduced LDL-C by 47.1% (49% placebo corrected) with a numerically slightly greater response (−53.5%) in patients with less than 2% of residual LDLR activity (121). Evinacumab had no effect on LDLR activity in lymphocytes from patients with HoFH, which remained similarly low in lymphocytes taken before and after evinacumab treatment (122). Derepression of endothelial lipase by inhibiting ANGPTL3 improves VLDL processing, generating VLDL remnants with reduced size and lipid content. These remnant particles are efficiently removed from the circulation by redundant remnant receptors. LDL-C decreases independently from LDLR activity as the production of LDL particles is reduced (123).

### ω-3 fatty acids

Although FH is characterized by high LDL-C, LDLR mutations may also impair the clearance of TRLs, increasing the concentration of remnant lipoproteins particularly in the postprandial state. Remnant lipoproteins are atherogenic and may also contribute to cardiovascular risk in patients with FH (89, 124, 125). Daily supplementation with 4 g of ω-3 fatty acid ethyl esters (46% eicosapentaenoic acid and 38% docosahexaenoic acid) during an 8-week open-label study reduced fasting TGs (−20%), apoB (−8%), VLDL-apoB-100 (−26%), and apoB-48 (−36%) in 20 intensively treated (mostly high-dose statins and ezetimibe) patients with HeFH. Postprandial remnant exposure was also reduced (total VLDL-apoB-100 area under curve −26%) (126). Although treatment with ω-3 fatty acids consistently reduce TRLs in clinical studies, most studies have not been able to demonstrate a reduction in cardiovascular outcomes. Cardiovascular outcomes were reduced in the JELIS (Japan EPA Lipid Intervention Study) and REDUCE-IT (Reduction of Cardiovascular Events with Icosapent Ethyl-Intervention Trial), both of which supplemented patients with eicosapentaenoic acid only rather than utilizing a mixture of ω-3 fatty acids. LDL-C had to be higher than 4.4 mmol/l or lower than 2.59 mmol/l for inclusion into JELIS and REDUCE-IT, respectively (127, 128). Concomitant lipid-lowering therapy was generally more intense in REDUCE-IT, which was published in 2019 compared with the JELIS study, which was published in 2007. Although there is no direct evidence of benefit of treatment with ω-3 fatty acids in patients with FH specifically, treatment with 2 g of icosapent ethyl twice daily should be considered in patients with adequately controlled LDL-C and residual hypertriglyceridemia. If LDL-C is not at target, further options to control LDL-C (see below) should be considered first.

## Conclusions

With modern lipid-lowering therapies, most patients with HeFH can achieve or come close to their LDL-C target. Earlier and more consistent treatment of HeFH from childhood should also reduce the need for treating to the extremely low targets required for patients with established CVD. For patients with HoFH, the largest breakthrough has come from therapies that bypass the LDLR, enabling clinicians to treat all patients with HoFH irrespective of their genotype with lipoprotein apheresis as last treatment option.

But even if LDL-C levels can now be starkly reduced in almost all patients with FH by combining statins, ezetimibe, PCSK9 inhibitors, and eventually apheresis, one hurdle still remains for patients with FH who concomitantly display elevated Lp(a) levels. No doubt that drugs inhibiting apo(a) expression that are currently into development will prove instrumental in that respect.

## Conflict of interest

A. G. reports personal fees for public speaking or consultancy support from Akcea Therapeutics, Amgen, Mylan, MSD, Novartis, Sanofi-Regeneron, and Unilever; G. L. reports research grants and personal fees from Sanofi-Regeneron, Amgen Inc, Nyrada Ltd, and AFFiRiS AG; D. B. reports grants for clinical trials and/or personal fees from Abbott, Akcea Therapeutics, Amgen, Amryt, AstraZeneca, Lib Therapeutics, Novartis, and Sanofi. K. C., J. G.-N., and C. M. have nothing to disclose.

## References

[1] Goldstein J.L., Dana S.E., Brunschede G.Y., Brown M.S. (1975). Genetic heterogeneity in familial hypercholesterolemia: evidence for two different mutations affecting functions of low-density lipoprotein receptor. Proc. Natl. Acad. Sci. U. S. A..

[2] Beheshti S.O., Madsen C.M., Varbo A., Nordestgaard B.G. (2020). Worldwide prevalence of familial hypercholesterolemia: meta-analyses of 11 million subjects. J. Am. Coll. Cardiol..

[3] Nordestgaard B.G., Chapman M.J., Humphries S.E., Ginsberg H.N., Masana L., Descamps O.S., Wiklund O., Hegele R.A., Raal F.J., Defesche J.C., Wiegman A., Santos R.D., Watts G.F., Parhofer K.G., Hovingh G.K. (2013). Familial hypercholesterolaemia is underdiagnosed and undertreated in the general population: guidance for clinicians to prevent coronary heart disease. Eur. Heart J..

[4] Cuchel M., Bruckert E., Ginsberg H.N., Raal F.J., Santos R.D., Hegele R.A., Kuivenhoven J.A., Nordestgaard B.G., Descamps O.S., Steinhagen-Thiessen E., Tybjærg-Hansen A., Watts G.F., Averna M., Boileau C., Borén J. (2014). Homozygous familial hypercholesterolaemia: new insights and guidance for clinicians to improve detection and clinical management. A position paper from the Consensus Panel on Familial Hypercholesterolaemia of the European Atherosclerosis Society. Eur. Heart J..

[5] D’Erasmo L., Minicocci I., Nicolucci A., Pintus P., Roeters Van Lennep J.E., Masana L., Mata P., Sánchez-Hernández R.M., Prieto-Matos P., Real J.T., Ascaso J.F., Lafuente E.E., Pocovi M., Fuentes F.J., Muntoni S. (2018). Autosomal recessive hypercholesterolemia: long-term cardiovascular outcomes. J. Am. Coll. Cardiol..

[6] Allen J.M., Thompson G.R., Myant N.B., Steiner R., Oakley C.M. (1980). Cadiovascular complications of homozygous familial hypercholesterolaemia. Br. Heart J..

[7] (1991). Risk of fatal coronary heart disease in familial hypercholesterolaemia. Scientific Steering Committee on behalf of the Simon Broome Register Group. BMJ.

[8] Bruckert E., Kalmykova O., Bittar R., Carreau V., Béliard S., Saheb S., Rosenbaum D., Bonnefont-Rousselot D., Thomas D., Emery C., Khoshnood B., Carrié A. (2017). Long-term outcome in 53 patients with homozygous familial hypercholesterolaemia in a single centre in France. Atherosclerosis.

[9] Thompson G.R., Blom D.J., Marais A.D., Seed M., Pilcher G.J., Raal F.J. (2018). Survival in homozygous familial hypercholesterolaemia is determined by the on-treatment level of serum cholesterol. Eur. Heart J..

[10] Nanchen D., Gencer B., Auer R., Räber L., Stefanini G.G., Klingenberg R., Schmied C.M., Cornuz J., Muller O., Vogt P., Jüni P., Matter C.M., Windecker S., Lüscher T.F., Mach F. (2015). Prevalence and management of familial hypercholesterolaemia in patients with acute coronary syndromes. Eur. Heart J..

[11] Amor-Salamanca A., Castillo S., Gonzalez-Vioque E., Dominguez F., Quintana L., Lluís-Ganella C., Escudier J.M., Ortega J., Lara-Pezzi E., Alonso-Pulpon L., Garcia-Pavia P. (2017). Genetically confirmed familial hypercholesterolemia in patients with acute coronary syndrome. J. Am. Coll. Cardiol..

[12] De Backer G., Besseling J., Chapman J., Hovingh G.K., Kastelein J.J.P., Kotseva K., Ray K., Reiner Ž., Wood D., De Bacquer D., EUROASPIRE Investigators (2015). Prevalence and management of familial hypercholesterolaemia in coronary patients: an analysis of EUROASPIRE IV, a study of the European Society of Cardiology. Atherosclerosis.

[13] Sabina B., Madsen C.M., Varbo A., Benn M., Nordestgaard B.G. (2018). Relationship of familial hypercholesterolemia and high low-density lipoprotein cholesterol to ischemic stroke. Circulation.

[14] Pérez de Isla L., Alonso R., Mata N., Saltijeral A., Muñiz O., Rubio-Marin P., Diaz-Diaz J.L., Fuentes F., de Andrés R., Zambón D., Galiana J., Piedecausa M., Aguado R., Mosquera D., Vidal J.I. (2016). Coronary heart disease, peripheral arterial disease, and stroke in familial hypercholesterolaemia: insights from the SAFEHEART Registry (Spanish Familial Hypercholesterolaemia Cohort Study). Arterioscler. Thromb. Vasc. Biol..

[15] van Aalst-Cohen E.S., Jansen A.C.M., Tanck M.W.T., Defesche J.C., Trip M.D., Lansberg P.J., Stalenhoef A.F.H., Kastelein J.J.P. (2006). Diagnosing familial hypercholesterolaemia: the relevance of genetic testing. Eur. Heart J..

[16] Williams R.R., Hunt S.C., Schumacher M.C., Hegele R.A., Leppert M.F., Ludwig E.H., Hopkins P.N. (1993). Diagnosing heterozygous familial hypercholesterolemia using new practical criteria validated by molecular genetics. Am. J. Cardiol..

[17] Akioyamen L.E., Genest J., Chu A., Inibhunu H., Ko D.T., Tu J.V. (2019). Risk factors for cardiovascular disease in heterozygous familial hypercholesterolemia: a systematic review and meta-analysis. J. Clin. Lipidol..

[18] Jansen A.C.M., van Aalst-Cohen E.S., Tanck M.W., Trip M.D., Lansberg P.J., Liem A.H., van Lennep H.W.O.R., Sijbrands E.J.G., Kastelein J.J.P. (2004). The contribution of classical risk factors to cardiovascular disease in familial hypercholesterolaemia: data in 2400 patients. J. Intern. Med..

[19] de Ferranti Sarah D., Rodday Angie Mae, Mendelson Michael M., Wong John B., Leslie Laurel K., Sheldrick R.C. (2016). Prevalence of Familial Hypercholesterolemia in the 1999 to 2012 United States National Health and Nutrition Examination Surveys (NHANES). Circulation.

[20] Chan D.C., Pang J., Hooper A.J., Burnett J.R., Bell D.A., Bates T.R., van Bockxmeer F.M., Watts G.F. (2015). Elevated lipoprotein(a), hypertension and renal insufficiency as predictors of coronary artery disease in patients with genetically confirmed heterozygous familial hypercholesterolemia. Int. J. Cardiol..

[21] Vlad C.-E., Foia L., Florea L., Costache I.-I., Covic A., Popescu R., Reurean-Pintilei D., Covic A. (2021). Evaluation of cardiovascular risk factors in patients with familial hypercholesterolemia from the North-Eastern area of Romania. Lipids Health Dis..

[22] Sun D., Cao Y.-X., You X.-D., Zhou B.-Y., Li S., Guo Y.-L., Zhang Y., Wu N.-Q., Zhu C.-G., Gao Y., Dong Q.-T., Liu G., Dong Q., Li J.-J. (2019). Clinical and genetic characteristics of familial hypercholesterolemia patients with type 2 diabetes. J. Endocrinol. Invest..

[23] Besseling J., Kastelein J.J.P., Defesche J.C., Hutten B.A., Hovingh G.K. (2015). Association between familial hypercholesterolemia and prevalence of type 2 diabetes mellitus. JAMA.

[24] Benn M., Watts G.F., Tybjaerg-Hansen A., Nordestgaard B.G. (2012). Familial hypercholesterolemia in the Danish general population: prevalence, coronary artery disease, and cholesterol-lowering medication. J. Clin. Endocrinol. Metab..

[25] Leigh S., Futema M., Whittall R., Taylor-Beadling A., Williams M., den Dunnen J.T., Humphries S.E. (2017). The UCL low-density lipoprotein receptor gene variant database: pathogenicity update. J. Med. Genet..

[26] Vrablik M., Tichý L., Freiberger T., Blaha V., Satny M., Hubacek J.A. (2020). Genetics of familial hypercholesterolemia: new insights. Front. Genet..

[27] Hobbs H.H., Russell D.W., Brown M.S., Goldstein J.L. (1990). The LDL receptor locus in familial hypercholesterolemia: Mutational Analysis of a Membrane Protein. Annu. Rev. Genet..

[28] Goldstein J.L., Brown M.S. (1974). Binding and degradation of low density lipoproteins by cultured human fibroblasts comparison of cells from a normal subject and from a patient with homozygous familial hypercholesterolemia. J. Biol. Chem..

[29] Chan P., Jones C., Lafrenière R., Parsons H.G. (1997). Surface expression of low density lipoprotein receptor in EBV-transformed lymphocytes: characterization and use for studying familial hypercholesterolemia. Atherosclerosis.

[30] Raungaard B., Heath F., Brorholt-Petersen J.U., Jensen H.K., Faergeman O. (1998). Flow cytometry with a monoclonal antibody to the low density lipoprotein receptor compared with gene mutation detection in diagnosis of heterozygous familial hypercholesterolemia. Clin. Chem..

[31] Romano M., Taranto M.D.D., Mirabelli P., D’Agostino M.N., Iannuzzi A., Marotta G., Gentile M., Raia M., Noto R.D., Vecchio L.D., Rubba P., Fortunato G. (2011). An improved method on stimulated T-lymphocytes to functionally characterize novel and known LDLR mutations. J. Lipid Res..

[32] Tada H., Kawashiri M., Noguchi T., Mori M., Tsuchida M., Takata M., Nohara A., Inazu A., Kobayashi J., Yachie A., Mabuchi H., Yamagishi M. (2009). A novel method for determining functional LDL receptor activity in familial hypercholesterolemia: Application of the CD3/CD28 assay in lymphocytes. Clinica Chim. Acta.

[33] Chan P., Huang T.-Y., Tomlinson B., Lee C., Lee Y.-S. (1997). Short-term safety and efficacy of low-dose simvastatin in elderly patients with hypertensive hypercholesterolemia and fasting hyperinsulinemia. J. Clin. Pharmacol..

[34] Sakuma N., Iwata S., Ichikawa T., Fujinami T. (1992). Assessment of functional low-density-lipoprotein receptors on lymphocytes by a simplified method using culture medium with lipoprotein-free fetal calf serum and pravastatin. Clin. Biochem..

[35] Etxebarria A., Benito-Vicente A., Alves A.C., Ostolaza H., Bourbon M., Martin C. (2014). Advantages and versatility of fluorescence-based methodology to characterize the functionality of LDLR and class mutation assignment. PLoS One.

[36] Silva S., Alves A.C., Patel D., Malhó R., Soutar A.K., Bourbon M. (2012). In vitro functional characterization of missense mutations in the LDLR gene. Atherosclerosis.

[37] Wang S., Mao Y., Narimatsu Y., Ye Z., Tian W., Goth C.K., Lira-Navarrete E., Pedersen N.B., Benito-Vicente A., Martin C., Uribe K.B., Hurtado-Guerrero R., Christoffersen C., Seidah N.G., Nielsen R. (2019). Site-specific O-glycosylation of members of the low-density lipoprotein receptor superfamily enhances ligand interactions. J. Biol. Chem..

[38] Etxebarria A., Benito-Vicente A., Palacios L., Stef M., Cenarro A., Civeira F., Ostolaza H., Martin C. (2015). Functional characterization and classification of frequent low-density lipoprotein receptor variants. Hum. Mutat..

[39] Innerarity T.L., Weisgraber K.H., Arnold K.S., Mahley R.W., Krauss R.M., Vega G.L., Grundy S.M. (1987). Familial defective apolipoprotein B-100: low density lipoproteins with abnormal receptor binding. Proc. Natl. Acad. Sci. U.S.A..

[40] Alves A.C., Etxebarria A., Soutar A.K., Martin C., Bourbon M. (2014). Novel functional APOB mutations outside LDL-binding region causing familial hypercholesterolaemia. Hum. Mol. Genet..

[41] Alves A.C., Benito-Vicente A., Medeiros A.M., Reeves K., Martin C., Bourbon M. (2018). Further evidence of novel APOB mutations as a cause of familial hypercholesterolaemia. Atherosclerosis.

[42] Esfahani M., Scerbo L., Devlin T.M. (1984). A requirement for cholesterol and its structural features for a human macrophage-like cell line. J. Cell Biochem..

[43] Huang S., Henry L., Ho Y.K., Pownall H.J., Rudenko G. (2010). Mechanism of LDL binding and release probed by structure-based mutagenesis of the LDL receptor. J. Lipid Res..

[44] Abifadel M., Varret M., Rabès J.-P., Allard D., Ouguerram K., Devillers M., Cruaud C., Benjannet S., Wickham L., Erlich D., Derré A., Villéger L., Farnier M., Beucler I., Bruckert E. (2003). Mutations in PCSK9 cause autosomal dominant hypercholesterolemia. Nat. Genet..

[45] Lambert G., Sjouke B., Choque B., Kastelein J.J.P., Hovingh G.K. (2012). The PCSK9 decade Thematic Review Series: new lipid and lipoprotein targets for the treatment of cardiometabolic diseases. J. Lipid Res..

[46] Dron J.S., Hegele R.A. (2017). Complexity of mechanisms among human proprotein convertase subtilisin–kexin type 9 variants. Curr. Opin. Lipidol..

[47] Huijgen R., Blom D.J., Hartgers M.L., Chemello K., Benito-Vicente A., Uribe K.B., Behardien Z., Blackhurst D.M., Brice B.C., Defesche J.C., de Jong A.G., Jooste R.J., Ratanjee B.D., Solomon G.A.E., Wolmarans K.H. (2021). Novel PCSK9 (proprotein convertase subtilisin kexin type 9) variants in patients with familial hypercholesterolemia from Cape Town. Arterioscler. Thromb. Vasc. Biol..

[48] Cameron J., Holla Ø.L., Ranheim T., Kulseth M.A., Berge K.E., Leren T.P. (2006). Effect of mutations in the PCSK9 gene on the cell surface LDL receptors. Hum. Mol. Genet..

[49] Sun X.-M., Eden E.R., Tosi I., Neuwirth C.K., Wile D., Naoumova R.P., Soutar A.K. (2005). Evidence for effect of mutant PCSK9 on apolipoprotein B secretion as the cause of unusually severe dominant hypercholesterolaemia. Hum. Mol. Genet..

[50] Di Taranto M.D., Benito-Vicente A., Giacobbe C., Uribe K.B., Rubba P., Etxebarria A., Guardamagna O., Gentile M., Martín C., Fortunato G. (2017). Identification and in vitro characterization of two new PCSK9 Gain of Function variants found in patients with Familial Hypercholesterolemia. Sci. Rep..

[51] Alves A.C., Etxebarria A., Medeiros A.M., Benito-Vicente A., Thedrez A., Passard M., Croyal M., Martin C., Lambert G., Bourbon M. (2015). Characterization of the first PCSK9 gain of function homozygote. J. Am. Coll. Cardiol..

[52] Sánchez-Hernández R.M., Di Taranto M.D., Benito-Vicente A., Uribe K.B., Lamiquiz-Moneo I., Larrea-Sebal A., Jebari S., Galicia-Garcia U., Nóvoa F.J., Boronat M., Wägner A.M., Civeira F., Martín C., Fortunato G. (2019). The Arg499His gain-of-function mutation in the C-terminal domain of PCSK9. Atherosclerosis.

[53] Garcia C.K., Wilund K., Arca M., Zuliani G., Fellin R., Maioli M., Calandra S., Bertolini S., Cossu F., Grishin N., Barnes R., Cohen J.C., Hobbs H.H. (2001). Autosomal recessive hypercholesterolemia caused by mutations in a putative LDL receptor adaptor protein. Science.

[54] Maurer M.E., Cooper J.A. (2006). The adaptor protein Dab2 sorts LDL receptors into coated pits independently of AP-2 and ARH. J. Cell Sci..

[55] Eden E.R., Patel D.D., Sun X.-M., Burden J.J., Themis M., Edwards M., Lee P., Neuwirth C., Naoumova R.P., Soutar A.K. (2002). Restoration of LDL receptor function in cells from patients with autosomal recessive hypercholesterolemia by retroviral expression of *ARH1*. J. Clin. Invest..

[56] Wilund K.R., Yi M., Campagna F., Arca M., Zuliani G., Fellin R., Ho Y.-K., Garcia J.V., Hobbs H.H., Cohen J.C. (2002). Molecular mechanisms of autosomal recessive hypercholesterolemia. Hum. Mol. Genet..

[57] Thedrez A., Sjouke B., Passard M., Prampart-Fauvet S., Guédon A., Croyal M., Dallinga-Thie G., Peter J., Blom D., Ciccarese M., Cefalù Angelo B., Pisciotta L., Santos Raul D., Averna M., Raal F. (2016). Proprotein convertase subtilisin kexin type 9 inhibition for autosomal recessive hypercholesterolemia—brief report. Arteriosclerosis, Thromb. Vasc. Biol..

[58] Nikkilä K., Åberg F., Isoniemi H. (2014). Transmission of LDLR mutation from donor through liver transplantation resulting in hypercholesterolemia in the recipient. Am. J. Transplant..

[59] Lingenhel A., Kraft H.G., Kotze M., Peeters A.V., Kronenberg F., Kruse R., Utermann G. (1998). Concentrations of the atherogenic Lp(a) are elevated in familial hypercholesterolaemia: a sib pair and family analysis. Eur. J. Hum. Genet..

[60] Kraft H.G., Lingenhel A., Raal F.J., Hohenegger M., Utermann G. (2000). Lipoprotein(a) in homozygous familial hypercholesterolemia. Arterioscler. Thromb. Vasc. Biol..

[61] van der Hoek Y.Y., Lingenhel A., Kraft H.G., Defesche J.C., Kastelein J.J., Utermann G. (1997). Sib-pair analysis detects elevated Lp(a) levels and large variation of Lp(a) concentration in subjects with familial defective ApoB. J. Clin. Invest..

[62] Tada H., Kawashiri M., Yoshida T., Teramoto R., Nohara A., Konno T., Inazu A., Mabuchi H., Yamagishi M., Hayashi K. (2016). Lipoprotein(a) in familial hypercholesterolemia with proprotein convertase subtilisin/kexin type 9 (*PCSK9*) gain-of-function mutations. Circ. J..

[63] Romagnuolo R., Scipione C.A., Boffa M.B., Marcovina S.M., Seidah N.G., Koschinsky M.L. (2015). Lipoprotein(a) catabolism is regulated by proprotein convertase subtilisin/kexin type 9 through the low density lipoprotein receptor. J. Biol. Chem..

[64] Villard E.F., Thedrez A., Blankenstein J., Croyal M., Tran T.-T.-T., Poirier B., Le Bail J.-C., Illiano S., Nobécourt E., Krempf M., Blom D.J., Marais A.D., Janiak P., Muslin A.J., Guillot E. (2016). PCSK9 modulates the secretion but not the cellular uptake of lipoprotein(a) ex vivo: an effect blunted by alirocumab. JACC Basic Transl. Sci..

[65] Chemello K., Beeské S., Tran T.T.T., Blanchard V., Villard E.F., Poirier B., Bail J.-C.L., Dargazanli G., Ho-Van-Guimbal S., Boulay D., Bergis O., Pruniaux M.-P., Croyal M., Janiak P., Guillot E. (2020). Lipoprotein(a) cellular uptake ex vivo and hepatic capture in vivo is insensitive to PCSK9 inhibition with alirocumab. JACC Basic Transl. Sci..

[66] Croyal M., Tran T.-T.-T., Blanchard R.H., Le Bail J.-C., Villard E.F., Poirier B., Aguesse A., Billon-Crossouard S., Ramin-Mangata S., Blanchard V., Nativel B., Chemello K., Khantalin I., Thedrez A. (2018). PCSK9 inhibition with alirocumab reduces lipoprotein(a) levels in nonhuman primates by lowering apolipoprotein(a) production rate. Clin. Sci. (Lond)..

[67] Rader D.J., Mann W.A., Cain W., Kraft H.G., Usher D., Zech L.A., Hoeg J.M., Davignon J., Lupien P., Grossman M. (1995). The low density lipoprotein receptor is not required for normal catabolism of Lp(a) in humans. J. Clin. Invest..

[68] Reyes-Soffer G., Pavlyha M., Ngai C., Thomas T., Holleran S., Ramakrishnan R., Karmally W., Nandakumar R., Fontanez N., Obunike J., Marcovina S.M., Lichtenstein A.H., Matthan N.R., Matta J., Maroccia M. (2017). Effects of PCSK9 inhibition with alirocumab on lipoprotein metabolism in healthy humans. Circulation.

[69] Watts G.F., Chan D.C., Somaratne R., Wasserman S.M., Scott R., Marcovina S.M., Barrett P.H.R. (2018). Controlled study of the effect of proprotein convertase subtilisin-kexin type 9 inhibition with evolocumab on lipoprotein(a) particle kinetics. Eur. Heart J..

[70] Khera A.V., Everett B.M., Caulfield M.P., Hantash F.M., Wohlgemuth J., Ridker P.M., Mora S. (2014). Lipoprotein(a) concentrations, rosuvastatin therapy, and residual vascular risk: an analysis from the JUPITER trial. Circulation.

[71] Kronenberg F., Utermann G. (2013). Lipoprotein(a): resurrected by genetics. J. Intern. Med..

[72] Prassl R., Schuster B., Abuja P.M., Zechner M., Kostner G.M., Laggner P. (1995). A comparison of structure and thermal behavior in human plasma lipoprotein(a) and low-density lipoprotein. Calorimetry and small-angle X-ray scattering. Biochemistry.

[73] Phillips M.L., Lembertas A.V., Schumaker V.N., Lawn R.M., Shire S.J., Zioncheck T.F. (1994). Electron-microscopic and hydrodynamic characterization of recombinant apolipoprotein (a) and its association with LDL. Chem. Phys. Lipids.

[74] García-Nafría J., Tate C.G. (2020). Cryo-Electron microscopy: moving beyond X-ray crystal structures for drug receptors and drug development. Annu. Rev. Pharmacol. Toxicol..

[75] Ren G., Rudenko G., Ludtke S.J., Deisenhofer J., Chiu W., Pownall H.J. (2010). Model of human low-density lipoprotein and bound receptor based on CryoEM. Proc. Natl. Acad. Sci. U.S.A..

[76] Langsted A., Kamstrup P.R., Benn M., Tybjærg-Hansen A., Nordestgaard B.G. (2016). High lipoprotein(a) as a possible cause of clinical familial hypercholesterolaemia: a prospective cohort study. Lancet Diabetes Endocrinol..

[77] Langsted A., Nordestgaard B.G., Benn M., Tybjærg-Hansen A., Kamstrup P.R. (2016). PCSK9 R46L loss-of-function mutation reduces lipoprotein(a), LDL cholesterol, and risk of aortic valve stenosis. J. Clin. Endocrinol. Metab..

[78] Trinder M., DeCastro M.L., Azizi H., Cermakova L., Jackson L.M., Frohlich J., Mancini G.B.J., Francis G.A., Brunham L.R. (2020). Ascertainment bias in the association between elevated lipoprotein(a) and familial hypercholesterolemia. J. Am. Coll. Cardiol..

[79] Clarke R., Peden J.F., Hopewell J.C., Kyriakou T., Goel A., Heath S.C., Parish S., Barlera S., Franzosi M.G., Rust S., Bennett D., Silveira A., Malarstig A., Green F.R., Lathrop M. (2009). Genetic variants associated with Lp(a) lipoprotein level and coronary disease. N. Engl. J. Med..

[80] Hogue J.-C., Lamarche B., Gaudet D., Tremblay A.J., Després J.-P., Bergeron J., Gagné C., Couture P. (2007). Association of heterozygous familial hypercholesterolemia with smaller HDL particle size. Atherosclerosis.

[81] Galvan A.Q., Santoro D., Natali A., Sampietro T., Boni C., Masoni A., Buzzigoli G., Ferrannini E. (1993). Insulin sensitivity in familial hypercholesterolemia. Metabolism.

[82] Gaudet D., Vohl M.C., Perron P., Tremblay G., Gagné C., Lesiège D., Bergeron J., Moorjani S., Després J.P. (1998). Relationships of abdominal obesity and hyperinsulinemia to angiographically assessed coronary artery disease in men with known mutations in the LDL receptor gene. Circulation.

[83] Bellanger N., Orsoni A., Julia Z., Fournier N., Frisdal E., Duchene E., Bruckert E., Carrie A., Bonnefont-Rousselot D., Pirault J., Saint-Charles F., Chapman M.J., Lesnik P., Le Goff W. (2011). Atheroprotective reverse cholesterol transport pathway is defective in familial hypercholesterolemia. Arteriosclerosis, Thromb. Vasc. Biol..

[84] Guerin M. (2012). Reverse cholesterol transport in familial hypercholesterolemia. Curr. Opin. Lipidol..

[85] Martinez L.R.C., Santos R.D., Miname M.H., Deus D.F., Lima E.S., Maranhão R.C. (2013). Transfer of lipids to high-density lipoprotein (HDL) is altered in patients with familial hypercholesterolemia. Metabolism.

[86] Ganjali S., Momtazi A.A., Banach M., Kovanen P.T., Stein E.A., Sahebkar A. (2017). HDL abnormalities in familial hypercholesterolemia: Focus on biological functions. Prog. Lipid Res..

[87] Frénais R., Ouguerram K., Maugeais C., Marchini J.S., Benlian P., Bard J.M., Magot T., Krempf M. (1999). Apolipoprotein A-I kinetics in heterozygous familial hypercholesterolemia: a stable isotope study. J. Lipid Res..

[88] Bouhali T., Brisson D., St-Pierre J., Tremblay G., Perron P., Laprise C., Vohl M.-C., Vissers M.N., Hutten B.A., Després J.-P., Kastelein J.J.P., Gaudet D. (2008). Low plasma adiponectin exacerbates the risk of premature coronary artery disease in familial hypercholesterolemia. Atherosclerosis.

[89] Chan D.C., Watts G.F. (2012). Postprandial lipoprotein metabolism in familial hypercholesterolemia: thinking outside the box. Metabolism.

[90] Nunes V.S., Cazita P.M., Catanozi S., Nakandakare E.R., Quintão E.C.R. (2020). Cholesterol metabolism in mice models of genetic hypercholesterolemia. J. Physiol. Biochem..

[91] Sniderman A.D., Tsimikas S., Fazio S. (2014). The Severe hypercholesterolemia phenotype: clinical diagnosis, management, and emerging therapies. J. Am. Coll. Cardiol..

[92] Dietschy J.M., Turley S.D., Spady D.K. (1993). Role of liver in the maintenance of cholesterol and low density lipoprotein homeostasis in different animal species, including humans. J. Lipid Res..

[93] Ouguerram K., Chetiveaux M., Zair Y., Costet P., Abifadel M., Varret M., Boileau C., Magot T., Krempf M. (2004). Apolipoprotein B100 metabolism in autosomal-dominant hypercholesterolemia related to mutations in PCSK9. Arterioscler. Thromb. Vasc. Biol..

[94] Drouin-Chartier J.-P., Hogue J.-C., Tremblay A.J., Bergeron J., Lamarche B., Couture P. (2017). The elevation of plasma concentrations of apoB-48-containing lipoproteins in familial hypercholesterolemia is independent of PCSK9 levels. Lipids Health Dis..

[95] Horton J.D., Cohen J.C., Hobbs H.H. (2009). PCSK9: a convertase that coordinates LDL catabolism. J. Lipid Res..

[96] Wiegman A., Gidding S.S., Watts G.F., Chapman M.J., Ginsberg H.N., Cuchel M., Ose L., Averna M., Boileau C., Borén J., Bruckert E., Catapano A.L., Defesche J.C., Descamps O.S., Hegele R.A. (2015). Familial hypercholesterolaemia in children and adolescents: gaining decades of life by optimizing detection and treatment. Eur. Heart J..

[97] Versmissen J., Oosterveer D.M., Yazdanpanah M., Defesche J.C., Basart D.C.G., Liem A.H., Heeringa J., Witteman J.C., Lansberg P.J., Kastelein J.J.P., Sijbrands E.J.G. (2008). Efficacy of statins in familial hypercholesterolaemia: a long term cohort study. BMJ.

[98] Besseling J., Hovingh G.K., Huijgen R., Kastelein J.J.P., Hutten B.A. (2016). Statins in familial hypercholesterolemia: consequences for coronary artery disease and all-cause mortality. J. Am. Coll. Cardiol..

[99] Luirink I.K., Wiegman A., Kusters D.M., Hof M.H., Groothoff J.W., de Groot E., Kastelein J.J.P., Hutten B.A. (2019). 20-year follow-up of statins in children with familial hypercholesterolemia. N. Engl. J. Med..

[100] Raal Frederick J., Pilcher Gillian J., Panz Vanessa R., van Deventer Hendrick E., Brice Brigitte C., Blom Dirk J., Marais A. David. (2011). Reduction in mortality in subjects with homozygous familial hypercholesterolemia associated with advances in lipid-lowering therapy. Circulation.

[101] Wierzbicki A.S., Lumb P.J., Semra Y.K., Crook M.A. (1998). High-dose atorvastatin therapy in severe heterozygous familial hypercholesterolaemia. QJM.

[102] David M.A., Firth J.C., Bateman M.E., Byrnes P., Martens C., Mountney J. (1997). Atorvastatin: an effective lipid-modifying agent in familial hypercholesterolemia. Arterioscler. Thromb. Vasc. Biol..

[103] Stein E.A., Strutt K., Southworth H., Diggle P.J., Miller E. (2003). Comparison of rosuvastatin versus atorvastatin in patients with heterozygous familial hypercholesterolemia. Am. J. Cardiol..

[104] Marais A.D., Blom D.J., Firth J.C. (2002). Statins in homozygous familial hypercholesterolemia. Curr. Atheroscler. Rep..

[105] Di Taranto M.D., Giacobbe C., Buonaiuto A., Calcaterra I., Palma D., Maione G., Iannuzzo G., Di Minno M.N.D., Rubba P., Fortunato G. (2020). A real-world experience of clinical, biochemical and genetic assessment of patients with homozygous familial hypercholesterolemia. J. Clin. Med..

[106] Davis H.R.J., Tershakovec A.M., Tomassini J.E., Musliner T. (2011). Intestinal sterol transporters and cholesterol absorption inhibition. Curr. Opin. Lipidol..

[107] Kastelein J., Akdim F., Stroes E., Zwinderman A., Bots M., Stalenhoef A., Visseren F., Sijbrands E., Stein E., Gaudet D., Duivenvoorden R., Veltri E., Marais D., Groot E. (2008). Simvastatin with or without ezetimibe in familial hypercholesterolemia. N. Engl. J. Med..

[108] Gagné C., Gaudet D., Bruckert E., Ezetimibe Study Group (2002). Efficacy and safety of ezetimibe coadministered with atorvastatin or simvastatin in patients with homozygous familial hypercholesterolemia. Circulation.

[109] Marais A.D., Kim J.B., Wasserman S.M., Lambert G. (2015). PCSK9 inhibition in LDL cholesterol reduction: Genetics and therapeutic implications of very low plasma lipoprotein levels. Pharmacol. Ther..

[110] Kastelein J.J.P., Ginsberg H.N., Langslet G., Hovingh G.K., Ceska R., Dufour R., Blom D., Civeira F., Krempf M., Lorenzato C., Zhao J., Pordy R., Baccara-Dinet M.T., Gipe D.A., Geiger M.J., Farnier M. (2015). ODYSSEY FH I and FH II: 78 week results with alirocumab treatment in 735 patients with heterozygous familial hypercholesterolaemia. Eur. Heart J..

[111] Raal F.J., Stein E.A., Dufour R., Turner T., Civeira F., Burgess L., Langslet G., Scott R., Olsson A.G., Sullivan D., Hovingh G.K., Cariou B., Gouni-Berthold I., Somaratne R., Bridges I. (2015). PCSK9 inhibition with evolocumab (AMG 145) in heterozygous familial hypercholesterolaemia (RUTHERFORD-2): a randomised, double-blind, placebo-controlled trial. Lancet.

[112] Ginsberg H.N., Rader D.J., Raal F.J., Guyton J.R., Baccara-Dinet M.T., Lorenzato C., Pordy R., Stroes E. (2016). Efficacy and safety of alirocumab in patients with heterozygous familial hypercholesterolemia and LDL-C of 160 mg/dl or higher. Cardiovasc. Drugs Ther..

[113] Stein E.A., Honarpour N., Wasserman S.M., Xu F., Scott R., Raal F.J. (2013). Effect of the proprotein convertase subtilisin/kexin 9 monoclonal antibody, AMG 145, in homozygous familial hypercholesterolemia. Circulation.

[114] Raal F.J., Honarpour N., Blom D.J., Hovingh G.K., Xu F., Scott R., Wasserman S.M., Stein E.A. (2015). Inhibition of PCSK9 with evolocumab in homozygous familial hypercholesterolaemia (TESLA Part B): a randomised, double-blind, placebo-controlled trial. Lancet.

[115] Thedrez A., Blom D.J., Ramin-Mangata S., Blanchard V., Croyal M., Chemello K., Nativel B., Pichelin M., Cariou B., Bourane S., Tang L., Farnier M., Raal F.J., Lambert G. (2018). Homozygous FH patients with identical mutations variably express the LDL receptor: implications for the efficacy of evolocumab. Arterioscler. Thromb. Vasc. Biol..

[116] Raal F.J., Kallend D., Ray K.K., Turner T., Koenig W., Wright R.S., Wijngaard P.L.J., Curcio D., Jaros M.J., Leiter L.A., Kastelein J.J.P. (2020). Inclisiran for the treatment of heterozygous familial hypercholesterolemia. N. Engl. J. Med..

[117] Cuchel M., Meagher E., du Toit T.H., Blom D., Marais A., Hegele R., Averna M., Sirtori C., Shah P., Gaudet D., Stefanutti C., Vigna G., Du Plessis A., Propert K.J., Sasiela W., Bloedon L., Rader D. (2013). Efficacy and safety of a microsomal triglyceride transfer protein inhibitor in homozygous familial hypercholesterolemia. Lancet.

[118] Raal F.J., Santos R.D., Blom D.J., Marais A.D., Charng M.-J., Cromwell W.C., Lachmann R.H., Gaudet D., Tan J.L., Chasan-Taber S., Tribble D.L., Flaim J.D., Crooke S.T. (2010). Mipomersen, an apolipoprotein B synthesis inhibitor, for lowering of LDL cholesterol concentrations in patients with homozygous familial hypercholesterolaemia: a randomised, double-blind, placebo-controlled trial. Lancet.

[119] Minicocci I., Montali A., Robciuc M.R., Quagliarini F., Censi V., Labbadia G., Gabiati C., Pigna G., Sepe M.L., Pannozzo F., Lütjohann D., Fazio S., Jauhiainen M., Ehnholm C., Arca M. (2012). Mutations in the ANGPTL3 gene and familial combined hypolipidemia: a clinical and biochemical characterization. J. Clin. Endocrinol. Metab..

[120] Rosenson R.S., Burgess L.J., Ebenbichler C.F., Baum S.J., Stroes E.S.G., Ali S., Khilla N., Hamlin R., Pordy R., Dong Y., Son V., Gaudet D. (2020). Evinacumab in patients with refractory hypercholesterolemia. N. Engl. J. Med..

[121] Raal F.J., Rosenson R.S., Reeskamp L.F., Hovingh G.K., Kastelein J.J.P., Rubba P., Ali S., Banerjee P., Chan K.-C., Gipe D.A., Khilla N., Pordy R., Weinreich D.M., Yancopoulos G.D., Zhang Y. (2020). Evinacumab for homozygous familial hypercholesterolemia. N. Engl. J. Med..

[122] Banerjee P., Chan K.-C., Tarabocchia M., Benito-Vicente A., Alves A.C., Uribe K.B., Bourbon M., Skiba P.J., Pordy R., Gipe D.A., Gaudet D., Martin C. (2019). Functional analysis of ldlr (low-density lipoprotein receptor) variants in patient lymphocytes to assess the effect of evinacumab in homozygous familial hypercholesterolemia patients with a spectrum of LDLR activity. Arterioscler. Thromb. Vasc. Biol..

[123] Adam R.C., Mintah I.J., Alexa-Braun C.A., Shihanian L.M., Lee J.S., Banerjee P., Hamon S.C., Kim H.I., Cohen J.C., Hobbs H.H., Van Hout C., Gromada J., Murphy A.J., Yancopoulos G.D., Sleeman M.W. (2020). Angiopoietin-like protein 3 governs LDL-cholesterol levels through endothelial lipase-dependent VLDL clearance. J. Lipid Res..

[124] Kolovou G.D., Kostakou P.M., Anagnostopoulou K.K. (2011). Familial hypercholesterolemia and triglyceride metabolism. Int. J. Cardiol..

[125] Sandesara P.B., Virani S.S., Fazio S., Shapiro M.D. (2018). The forgotten lipids: triglycerides, remnant cholesterol, and atherosclerotic cardiovascular disease risk. Endocr. Rev..

[126] Chan D.C., Pang J., Barrett P.H.R., Sullivan D.R., Burnett J.R., van Bockxmeer F.M., Watts G.F. (2016). ω-3 Fatty acid ethyl esters diminish postprandial lipemia in familial hypercholesterolemia. J. Clin. Endocrinol. Metab..

[127] Yokoyama M., Origasa H., Matsuzaki M., Matsuzawa Y., Saito Y., Ishikawa Y., Oikawa S., Sasaki J., Hishida H., Itakura H., Kita T., Kitabatake A., Nakaya N., Sakata T., Shimada K. (2007). Effects of eicosapentaenoic acid on major coronary events in hypercholesterolaemic patients (JELIS): a randomised open-label, blinded endpoint analysis. Lancet.

[128] Bhatt D.L., Steg P.G., Miller M., Brinton E.A., Jacobson T.A., Ketchum S.B., Doyle R.T., Juliano R.A., Jiao L., Granowitz C., Tardif J.-C., Ballantyne C.M., REDUCE-IT Investigators (2019). Cardiovascular risk reduction with icosapent ethyl for hypertriglyceridemia. N. Engl. J. Med..

